# The Oregon Child Absenteeism Due to Respiratory Disease Study (ORCHARDS): Rationale, objectives, and design

**DOI:** 10.1111/irv.12920

**Published:** 2021-10-08

**Authors:** Jonathan L. Temte, Shari Barlow, Maureen Goss, Emily Temte, Cristalyne Bell, Cecilia He, Caroline Hamer, Amber Schemmel, Bradley Maerz, Lily Comp, Mitchell Arnold, Kimberly Breunig, Sarah Clifford, Erik Reisdorf, Peter Shult, Mary Wedig, Thomas Haupt, James Conway, Ronald Gangnon, Ashley Fowlkes, Amra Uzicanin

**Affiliations:** ^1^ Department of Family Medicine and Community Health University of Wisconsin School of Medicine and Public Health Madison Wisconsin USA; ^2^ Communicable Disease Division Wisconsin State Laboratory of Hygiene Madison Wisconsin USA; ^3^ Wisconsin Division of Public Health Wisconsin Department of Health Services Madison Wisconsin USA; ^4^ Department of Pediatrics, Division of Infectious Diseases University of Wisconsin School of Medicine and Public Health Madison Wisconsin USA; ^5^ Department of Biostatistics and Medical Informatics University of Wisconsin School of Medicine and Public Health Madison Wisconsin USA; ^6^ Division of Global Migration and Quarantine US Centers for Disease Control and Prevention Atlanta Georgia USA

**Keywords:** absenteeism, children, influenza, population surveillance, school

## Abstract

**Background:**

Influenza viruses pose significant disease burdens through seasonal outbreaks and unpredictable pandemics. Existing surveillance programs rely heavily on reporting of medically attended influenza (MAI). Continuously monitoring cause‐specific school absenteeism may identify local acceleration of seasonal influenza activity. The Oregon Child Absenteeism Due to Respiratory Disease Study (ORCHARDS; Oregon, WI) implements daily school‐based monitoring of influenza‐like illness‐specific student absenteeism (a‐ILI) in kindergarten through Grade 12 schools and assesses this approach for early detection of accelerated influenza and other respiratory pathogen transmission in schools and surrounding communities.

**Methods:**

Starting in September 2014, ORCHARDS combines automated reporting of daily absenteeism within six schools and home visits to school children with acute respiratory infection (ARI). Demographic, epidemiological, and symptom data are collected along with respiratory specimens. Specimens are tested for influenza and other respiratory viruses. Household members can opt into a supplementary household transmission study. Community comparisons are possible using a pre‐existing and highly effective influenza surveillance program, based on MAI at five family medicine clinics in the same geographical area.

**Results:**

Over the first 5 years, a‐ILI occurred on 6634 (0.20%) of 3,260,461 student school days. Viral pathogens were detected in 64.5% of 1728 children with ARI who received a home visit. Influenza was the most commonly detected virus, noted in 23.3% of ill students.

**Conclusion:**

ORCHARDS uses a community‐based design to detect influenza trends over multiple seasons and to evaluate the utility of absenteeism for early detection of accelerated influenza and other respiratory pathogen transmission in schools and surrounding communities.

**Abbreviations**
4k: 4‐year‐old pre‐kindergarten
a‐I: absenteeism due to illness
a‐ILI: absenteeism due to influenza‐like illness
ARI: acute respiratory infection
a‐TOT: all‐cause absenteeism
CDC: Centers for Disease Control and Prevention
FDA: Food and Drug Administration
FERPA: Family Educational Rights and Privacy Act
Flu: influenza
ILI: influenza‐like illness
IRB: institutional review board
IT: information technology
MAI: medically attended influenza
NP: nasopharyngeal
OP: oropharyngeal
ORCHARDS: Oregon Child Absenteeism Due to Respiratory Disease Study
OSD: Oregon School District
PCR: polymerase chain reaction
RedCap: Research Electronic Data Capture
RIDT: rapid influenza diagnostic test
RP: human RNAse P
RPP: respiratory pathogen panel
SIS: student information system
VTM: viral transport medium
W‐IISP: Wisconsin Influenza Incidence Surveillance Project
WIR: Wisconsin Immunization Registry
WSLH: Wisconsin State Laboratory of Hygiene

## 1 INTRODUCTION

Influenza viruses contribute to significant disease burdens and economic costs through annual seasonal outbreaks and unpredictable pandemics.^1^, ^2^, ^3^ Approaches for prevention and control of seasonal and pandemic influenza include vaccines, antiviral medications, and nonpharmaceutical interventions (NPIs), but their combined success is predicated on timely deployment. NPI, the first line of defense,^4^ hinges on early detection and recognition of outbreaks.^4^, ^5^, ^6^

Existing influenza surveillance programs rely on medical facilities to report cases of influenza‐like illness (ILI) and test‐confirmed influenza.^7^, ^8^ Even though influenza transmission among school‐aged children frequently precedes subsequent community transmission,^9^, ^10^ there have been no systematical evaluations of school‐based monitoring of influenza activity for complementing routine surveillance or serving as an early‐warning system for increased influenza activity in the wider community. Monitoring school absenteeism is feasible, as seasonal outbreaks occur between late fall and mid‐spring while schools are in session,^11^, ^12^ and most of the 13,588 school districts^13^ across the United States collect daily absenteeism data using electronic school information systems.^14^

During the 2009 influenza pandemic, there was high correlation (*r* = 0.92) reported between hospitalized influenza cases and school absenteeism due to ILI in one jurisdiction,^15^ likely enhanced by the short, intense nature of the outbreak, which amplified absenteeism related to ILI. Conversely, monitoring all‐cause absenteeism was less effective due to the multifactorial nature of absenteeism.^16^, ^17^, ^18^ The value of continuously monitoring cause‐specific absenteeism over the entire school year to identify local activity acceleration of seasonal influenza is not well understood.

**FIGURE 1:**
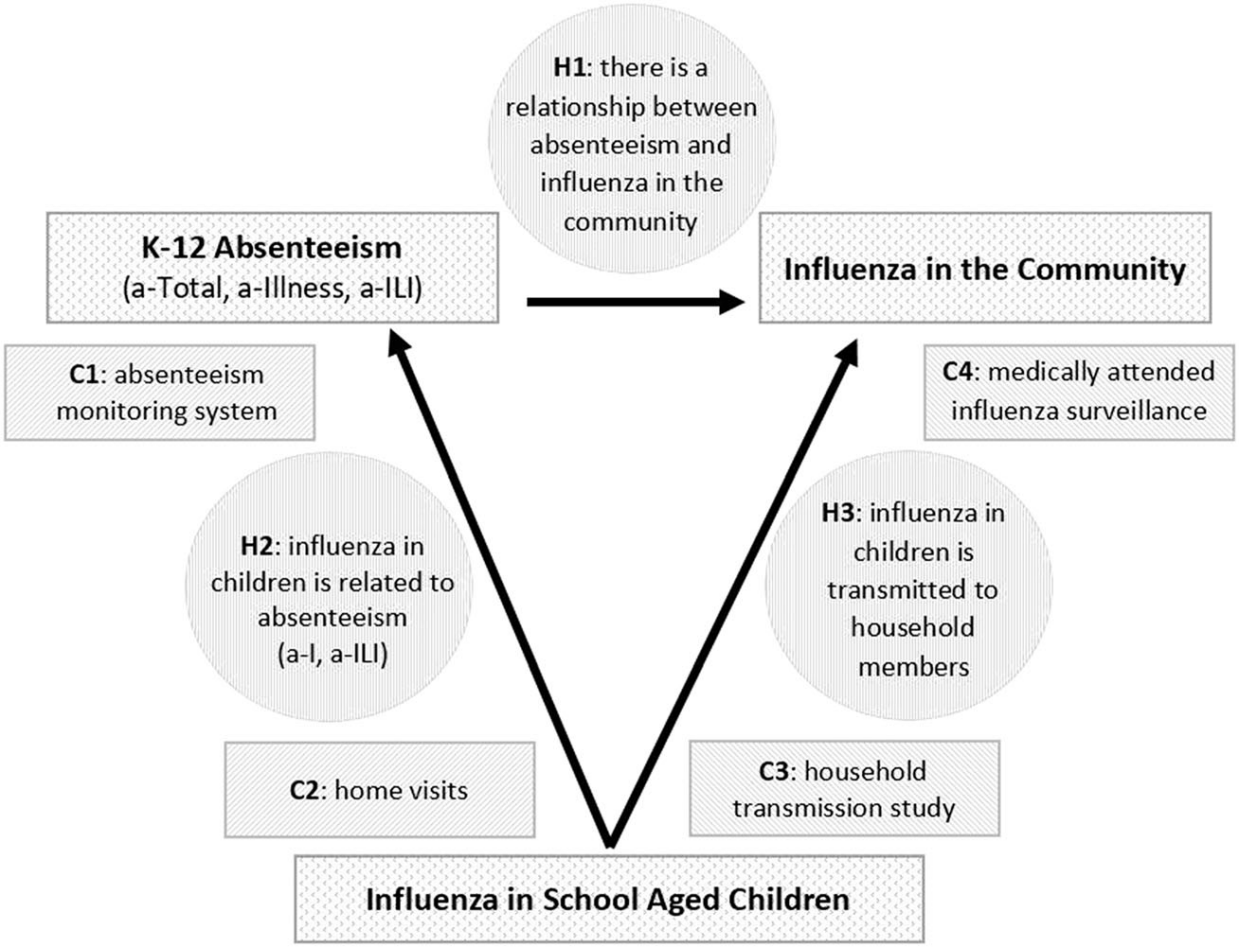
Theoretical framework of ORCHARDS demonstrating the relationships between influenza in school‐aged children, K‐12 school absenteeism, and medically attended influenza in the community. The relatedness of the four components (C1–C4) of ORCHARDS and the three primary hypotheses (H1–H3) are provided

The goal of Oregon Child Absenteeism Due to Respiratory Disease Study (ORCHARDS) is to develop and implement a system for daily school‐based monitoring of ILI‐specific student absenteeism in kindergarten through grade 12 (K‐12) schools and assess the system's usability for early detection of accelerated influenza and other respiratory pathogen transmission in schools and surrounding communities. The theoretical relationships between influenza infection in school‐aged children, K‐12 absenteeism, and influenza infection in the surrounding community, and study components and hypotheses are demonstrated in Figure 1.

## 2 METHODS

### 2.1 Location

The Oregon School District (OSD: www.oregonsd.org) encompasses the villages of Oregon and Brooklyn, decentralized subdivisions, and farms in Dane County, Wisconsin (latitude: 42.90 N, longitude: 89.43 W).^19^ The region experiences distinct temperate seasonality.

### 2.2 Community

The OSD population is estimated at 20,094 and is less racially and ethnically diverse, wealthier, and more formally educated than the United States average, while ages of individual family members and mean household size are similar (Table 1).^20^ The district is composed of 6 public schools enrolling 4091 students, including pre‐kindergarten students, in 2018–2019.^21^ There are 3 elementary schools (K‐4: 1503 students), 1 intermediate school (5–6: 623), 1 middle school (7–8: 596), and 1 high school (9–12: 1145).

**TABLE 1:** Demographic descriptions of the Oregon School District (OSD) as compared with Dane County, Wisconsin, and the United States

Demographic feature	OSD	Dane County	Wisconsin	United States
Total population	20,094	488,073	5,691,047	312,700,151
White [nH^a^] (%)	90.7	84.7	87.0	74.8
Black (%)	2.5	5.2	6.2	13.6
Am. Indian (%)	0.2	0.4	0.8	1.7
Asian (%)	1.9	4.7	2.3	5.6
Pacific Isl. (%)	0	0	0	0.4
Hispanic (%)	3.3	5.9	5.9	16.3
Ages (years)				
<5	5.9	6.2	6.2	6.5
5–19	21.3	18.7	17.2	20.4
20–44	33.6	39.2	36.0	33.6
45–64	30.3	25.7	27.7	26.4
65+	8.9	10.4	13.7	13.0
Household				
Mean size	2.69	2.33	2.43	2.58
<$15,000	4.4	10.9	6.1	13.4
$15,000–$35,000	10.5	18.6	33.1	22.3
$35,000–$75,000	33.0	32.0	46.0	32.5
>$75,000	52.0	38.4	14.8	31.7
Education				
< High school	4.1	2.1	9.9	14.4
High school/GED	29.7	20.6	33.3	28.5
Some college	30.3	27.0	30.5	28.9
Bachelor's	24.6	28.1	17.4	17.7
Post BA/BS	11.3	19.0	9.0	10.4

*Note*: Data from US Census.

^a^ nH, non‐Hispanic.

### 2.3 Timeframe

Initiation of ORCHARDS data collection occurred in phases. Absenteeism data collection commenced on September 2, 2014. Data collection from student home visits commenced on January 5, 2015. The household transmission substudy started on January 6, 2016. Medically attended influenza surveillance has been ongoing since October 2009.^8^ All components of ORCHARDS, including laboratory testing, have continued year‐around without interruption until present.

### 2.4 Absenteeism monitoring system

We modified an existing absenteeism reporting system. Parents report unscheduled absences using an automated telephone system, providing the student's name and the reason for absence, including symptoms if the child has a cold or flu‐like illness. Uniform messaging is on each school's absentee line:

Please inform us if your child has any flu‐like symptoms such as fever with cough, sneezing, chills, sore throat, body aches, fatigue, runny nose, and/or stuffy nose.

In the event that a student is absent without a report, OSD attendance staff make repeated efforts to contact the home or parents/guardians before the end of the day.

### 2.5 Absenteeism definition

Because of variability among schools in terms of the number of class periods for which a child can be absent and for simplicity/generalizability of electronic data retrieval, we consider a student absent for the entire day if absent for any part of a school day.

### 2.6 Types of absenteeism

All‐cause or total absenteeism (a‐TOT) is an absence for any reason. Absence due to illness (a‐I) is an absence due to any reported illness. Absence due to ILI (a‐ILI) is an a‐I for which ILI symptoms are reported.

### 2.7 a‐ILI definition

We considered established definitions for ILI^22^, ^23^ and used a simplified version of the Centers for Disease Control and Prevention (CDC) standard definition. ILI for ORCHARDS is defined as presence of fever and at least one respiratory symptom (cough, sore throat, nasal congestion, or runny nose).

### 2.8 Data system

OSD utilizes Infinite Campus®,^24^ a commercially available, electronic student information system (SIS), to track student attendance. This system allows attendance staff to identify a student, select a period, and select a reason for absence from a modifiable, drop‐down pick list. The OSD Information Technology (IT) staff added an option for “a‐ILI.”

### 2.9 Data extraction and secure file transfer

**FIGURE 2:**
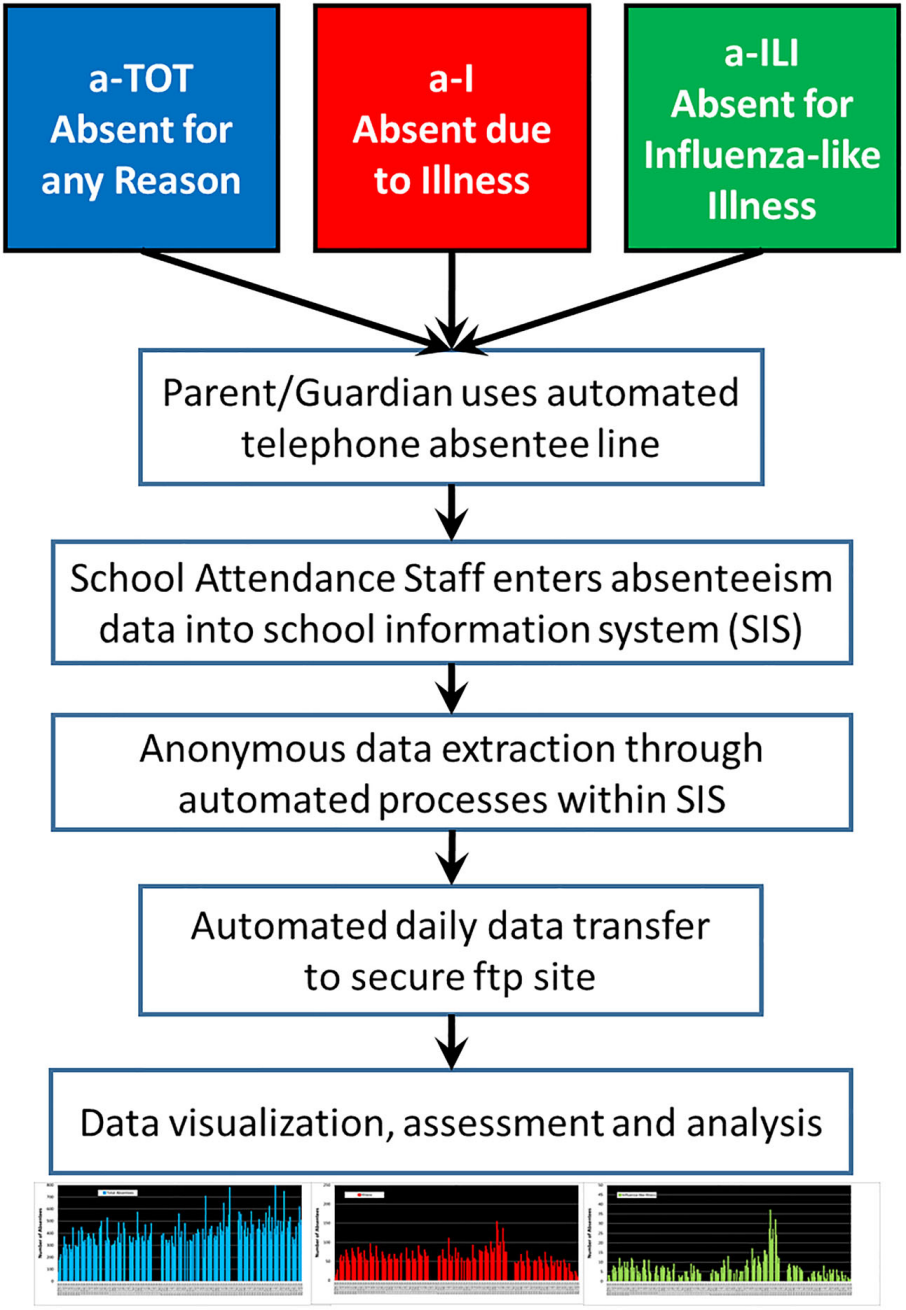
Flow diagram of absenteeism data from telephone reporting by parents/guardians, to entry into the student information system at the Oregon School District, to data transfer to the ORCHARDS research team

An automated process within Infinite Campus® extracts daily counts of absent individuals by school, grade, and absence type (a‐TOT, a‐I, and a‐ILI). Data contain no personal identifiers, are compliant with the Family Educational Rights and Privacy Act (FERPA: 20 U.S.C. § 1232g; 34 CFR Part 99),^25^ and are sent to a secure file transfer protocol (ftp) site at the University of Wisconsin Department of Family Medicine and Community Health. We track daily totals of absentee counts in each category. The data flow is illustrated in Figure 2.

### 2.10 School incentive

Each school receives $4000 per year to defray costs associated with IT support and effort by the attendance staff.

### 2.11 Assessment of influenza and absenteeism in children through home visits

#### 2.11.1 Contact and screening

Students are not required to be absent, and school does not need to be in session for home visits to occur. To comply with FERPA, interested parents/guardians call the study line to initiate a home visit. If a student meets the inclusion criteria, a 20‐min home visit occurs within 2 days of initial phone contact and within 7 days of symptom onset.

Inclusion criteria include:

1. student attends, or is eligible to attend (e.g., home schooled), a school within the OSD
2. has an illness characterized by ≥2 of 6 acute respiratory infection (ARI)/ILI symptoms (nasal discharge; nasal congestion; sneezing; sore throat; cough; and fever)
3. scores ≥2 points on the Jackson scale^26^, ^27^, ^28^

Exclusion criteria include:

1. illness onset ≥7 days before anticipated time of specimen collection
2. anatomical defect for which nasal specimen collection is contraindicated
3. student participated too recently (<7 days during peak influenza period and <30 days during other times, as determined by medically attended surveillance program)

If criteria are met, family members are invited to participate in an optional household transmission substudy. Participation is allowed even if individual members opt out or are unable to complete the entire study.

Inclusion criteria include:

1. individuals of any age/gender residing in the same household as ORCHARDS participant
2. able to provide appropriate consent/assent

Exclusion criteria include:

1. anatomical defect for which nasal sampling is contraindicated
2. household participated too recently (<7 days during peak influenza period and <30 days during other times, as determined by medically attended surveillance program)

#### 2.11.2 Acquiring informed consent/assent

Research coordinators obtain written informed consent from parents/guardians and/or adult students, and assent from younger students using forms tailored to reading levels based on age.

#### 2.11.3 Data collection

Demographic, epidemiologic, and symptom data are collected on an Acute Respiratory Infection and Influenza Surveillance Form (Data S1), based on a clinical instrument used since 2009 as part of the Wisconsin component of the Influenza Incidence Surveillance Project (W‐IISP).^8^ Accordingly, collected data are comparable with data from other surveillance systems.

#### 2.11.4 Respiratory specimen collection and handling

A nasal swab specimen is collected using a Puritan® Sterile Foam Tipped Applicator for rapid influenza diagnostic testing (RIDT). In addition, a nasopharyngeal (NP) or high oropharyngeal (OP) specimen is obtained using a Copan FLOQSwabs™ flocked swab. The NP/OP swab is placed into a 3.0‐ml Remel MicroTest™ M4RT® Transport viral transport medium (VTM) tube, placed into a small biohazard bag, and maintained at 2–8°C. Following processing for RIDT, the residual nasal swab is added to the VTM containing the NP/OP swab. A courier delivers the VTM tube to the Wisconsin State Laboratory of Hygiene (WSLH), usually within 24 h of collection.

#### 2.11.5 Incentives and feedback

Student participants receive a $20 gift card at the end of the home visit. Research coordinators call parents/guardians with RIDT results within 24 h (usually less than 4 h) of the home visit. Laboratory confirmed results are mailed to families within 2 weeks of a home visit and are accepted as documentation for an excused absence by the OSD.

### 2.12 Within‐household influenza transmission substudy

#### 2.12.1 Contact and screening

At the ORCHARDS home visit, recruited families receive a packet containing substudy information, instructions, consents/assents, and a collection kit for each household member. Each collection kit contains a data form, two nasal swabs, and two small biohazard bags, each of which contain a 3.0‐ml Remel MicroTest™ M4RT® VTM tube. VTM tubes and swabs for Day 0 and Day 7 are individually marked.

The research coordinator explains components of the family packet to all present household members and reviews consent/assent forms. A designated adult assures that all interested household participants sign the consent/assent forms prior to specimen collection. Household members collect their own specimens on Day 0 (within 24 h of home visit) and Day 7 (7 days after the initial collection). The timing (Day 7) for follow‐up data and specimen collection is based on prior influenza transmission studies.^29^, ^30^, ^31^

#### 2.12.2 Data collection

The ORCHARDS Household Study Form (Data S2) is used to collect data on:

1. Household composition: the number and characteristics of people sharing the household of an ORCHARDS participant. Information includes relationship to the ORCHARDS participant, age, gender, number of bedrooms in home, employment outside of home, school attendance, and daycare attendance.
2. Household member illness assessment on Day 0 (“Today” section): influenza vaccine status for the current season, and information about any ARI/ILI illnesses occurring over the past 7 days.
3. Household member illness assessment on Day 7, 1 week after the initial visit (“Follow‐up” section): information about any ARI/ILI illnesses occurring over the past 7 days.

#### 2.12.3 Respiratory specimen collection and handling

Household members are instructed on specimen collection using a Copan FLOQSwabs™ flocked midturbinate swab (January 6, 2016, through October 15, 2017) or an anterior nasal Puritan® Sterile Foam Tipped Applicator (starting on October 16, 2017). Each household participant collects the specimen without staff on Day 0 and Day 7. Upon retrieval, research coordinators review forms for completion and ensure specimens were collected and correspond with the appropriate labels.

#### 2.12.4 Household incentive for participation

Households completing the substudy receive a $50 gift card to local businesses.

### 2.13 Laboratory procedures

#### 2.13.1 Rapid influenza diagnostic test

We use the Quidel® Sofia® Influenza A + B fluorescent immunoassay^32^ for assessment of ORCHARDS participants, but not for household members.^32^ RIDT is performed at a nearby clinical facility within 6 h of specimen collection.

#### 2.13.2 Influenza rRT‐PCR

All specimens from students and household members (Day 0 and Day 7) are tested at WSLH for influenza A and B virus and Human RNase P (RP) using the in vitro diagnostic (IVD) Food and Drug Administration (FDA)‐approved CDC Human Influenza Virus Real‐time RT‐PCR Diagnostic Panel (Cat. # FluIVD03).^33^ The detection of RP, with a cycle threshold (Ct) value < 38, determines specimen adequacy for influenza polymerase chain reaction (PCR) testing.

#### 2.13.3 Multipathogen testing

All specimens from ORCHARDS students are tested for influenza A, influenza B, rhinovirus/enterovirus, adenovirus, parainfluenza virus,^1^, ^2^, ^3^, ^4^ seasonal coronavirus (HKU1, NL63, 229E, OC43), respiratory syncytial virus (A, B), human metapneumovirus and human bocavirus, and two atypical bacterial pathogens, *Chlamydia pneumoniae* and *Mycoplasma pneumoniae*, using the multiplexed PCR respiratory pathogen panel (RPP: Luminex NxTAG Respiratory Pathogen Panel).^34^

#### 2.13.4 Whole genome sequencing

For a subset of influenza‐positive subjects, we conduct next‐generation genome sequencing at the WSLH using the Illumina MiSeq™ platform. Sequence reads are trimmed using Trimmomatic with a 4‐bp sliding window quality score cutoff of Q30.^35^ The trimmed sequence reads are mapped against vaccine strain HA reference sequences using Geneious Version 11.0.5.^36^

#### 2.13.5 Specimen archiving

Aliquots of all residual specimens are archived at −70°C at WSLH.

#### 2.13.6 Validation of influenza vaccination

We validate influenza immunization status for all ORCHARDS students using the Wisconsin Immunization Registry (WIR).^37^ Vaccination status at the time of specimen collection is determined using criteria provided by the US Advisory Committee on Immunization Practices.^38^

#### 2.13.7 Data security and integrity

All identifying information on subjects is secured using a separate password‐protected security REDCap (Research Electronic Data Capture) database.^39^

#### 2.13.8 Community engagement and study promotion

ORCHARDS recruits using a reminder within the absenteeism reporting system. Information is provided at OSD registration each August and through e‐mails to OSD families. We employ flyers at community sites, presentations at community events, postcards, and the study website (www.fammed.wisc.edu/orchards/) and Facebook page (www.facebook.com/orchardstudy/).

#### 2.13.9 IRB and project oversight

This study was approved by the Human Subjects Committees of the Education and Social/Behavioral Sciences institutional review board (IRB) (initial approval on September 4, 2013) and the University of Wisconsin Health Sciences IRB (initial approval on December 5, 2013, with additional approvals as the protocol expanded and modified). The study is compliance with the Health Insurance Portability and Accountability Act of 1996 (HIPAA) and FERPA. The US Office of Management and Budget has approved all forms used in this study.

#### 2.13.10 Reference data—Assessment of influenza in the surrounding community

An independent surveillance system assesses medically attended influenza (MAI) at five family practice clinics within or adjoining the OSD. W‐IISP, sponsored by CDC and organized by the ORCHARDS team, has conducted active surveillance in medically attended patients with ARI/ILI since October 2009.^8^, ^40^ Specific clinic locations include Belleville, Oregon, Madison (two sites), and Verona. Together, the clinics recorded 412,752 ambulatory visits between June 29, 2014, and June 29, 2019, and assessed 4069 individual ARI patients.

NP/OP swabs are collected from a subset of ambulatory patients with ARI/ILI. Specimens are tested at the WSLH for influenza A and B using the rRT‐PCR^33^ and, for other viruses, using a multiplexed PCR RPP.^34^ Virological characterization is available for all (*n* = 4069) individual patient visits.

## 3 RESULTS

**FIGURE 3:**
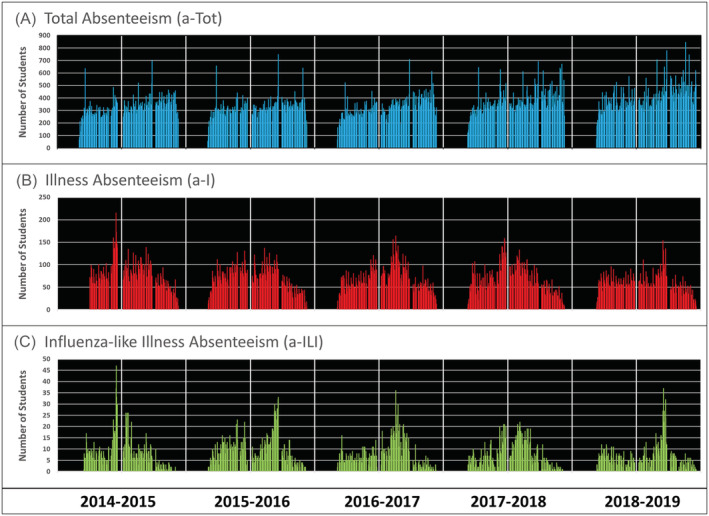
Daily counts of all absent students (a‐TOT: Panel A), students for whom an illness is reported (a‐I: Panel B), and students absent with influenza‐like illness (a‐ILI: Panel C) occurring over five consecutive school years at the Oregon School District, Wisconsin, USA, from September 2014 through June 2019. Vertical white bars demonstrate the timing of July 1 (thin bars) and January 1 (heavy bars)

The entire school district, including all schools, have participated since the initiation of ORCHARDS. Absenteeism data have been captured for all students, excluding pre‐kindergarten (for whom absenteeism is not recorded), since September 2014. The number of enrolled K‐12 students per year has increased from 3588 (2014/2015) to 3867 (2018/2019). Over the first 5 years of ORCHARDS, from September 2014 through June 2019, we evaluated 3,260,461 student days. Total absenteeism accounted for 301,427 (9.2%), a‐I for 58,126 (1.8%), and a‐ILI for 6634 (0.2%) of potential student days. The daily counts for each type of absenteeism, showing the variability, are depicted in Figure 3. Annual levels of absenteeism were similar across all 5 years (Table 2).

**TABLE 2:** Enrollment estimates and absenteeism by defined type for the Oregon School District, Oregon, Wisconsin: 2014–2019

	2014–2015	2015–2016	2016–2017	2017–2018	2018–2019
Total estimated enrollment^a^	3588	3713	3749	3828	3867
Total curricular days	176	177	175	176	174
Total possible student days	629,024	635,076	649,775	673,728	672,858
a‐TOT days (% of total)	56,477 (8.98%)	55,871 (8.80%)	56,511 (8.70%)	63,476 (9.42%)	69,092 (10.3%)
Average daily a‐TOT (std. dev.)	320.9 (78.4)	315.7 (79.2)	322.9 (80.4)	360.7 (96.3)	397.1 (106.2)
a‐I days (% of total)	12,024 (1.91%)	12,158 (1.91%)	11,922 (1.83%)	11,710 (1.74%)	10,312 (1.53%)
Average daily a‐I (std. dev.)	68.3 (39.0)	68.7 (27.0)	68.1 (27.0)	66.5 (30.1)	59.3 (21.7)
a‐ILI days (% of total)	1314 (0.21%)	1609 (0.25%)	1309 (0.20%)	1261 (0.19%)	1141 (0.17%)
Average daily a‐ILI (std. dev.)	7.5 (7.3)	9.1 (6.6)	7.5 (5.9)	7.2 (5.8)	6.6 (5.5)
Influenza vaccination rate in ORCHARDS participants	62.8%^b^	46.7%	48.8%	41.6%	54.7%

*Note*: The percentage of participants that were fully vaccinated against influenza is provided for each academic year.

^a^ Total estimated enrollment excluding 4k students (for whom absenteeism data are not submitted). Data extracted from Wisconsin Department of Public Instruction, Wisconsin Information System for Education data Dashboard: https://wisedash.dpi.wi.gov/Dashboard/portalHome.jsp.

^b^ Home visits did not start until January 5, 2015. The 2014–2015 vaccination rate is likely biased due to the late season sampling frame.

We completed 1728 home visits for children with ARI. Children ranged in age from 4 to 18 years (mean ± std. dev. = 9.9 ± 2.5 years). There were more male (57%) than female (43%) participants. Home visits occurred, on average, 56.3 ± 46.5 h after onset of symptoms. The number of home visits per day was positively correlated with a‐TOT (*r*_s_ = 0.20; *p* < 0.001), a‐I (*r*_s_ = 0.41; *p* < 0.001), and a‐ILI (*r*_s_ = 0.40; *p* < 0.001). Most children (79%) reported school absenteeism due to the current illness episode, and about half (853/1728: 49%) were fully vaccinated against influenza at the time of the home visit.

Pathogens were detected in 1115 (65%) specimens; the majority (99%) of these were viral. Codetections of viruses were noted in 66 students (6% of individuals with virus detections). Influenza was the most commonly detected virus, noted in 402/1728 (23%) students, followed by rhinovirus/enterovirus. The numbers of viruses detected are provided in Table 3.

**TABLE 3:** Detections of viruses from ORCHARDS participants through home visits (January 5, 2015, to June 30, 2019)

	Respiratory virus detected								
	Ad	CoV	FluA (H1)	FluA (H3)	FluB	hMPV	PIV	R/E	RSV
Number of detections	32	169	77	224	98	74	77	368	47
Percent	2.7	14.3	6.5	18.9	8.3	6.3	6.5	31.1	3.9

*Note*: The counts include dual detections (*n* = 64) and triple detections (*n* = 2). Not shown are data for influenza A (unable to subtype: *n* = 3), *Mycoplasma pneumoniae* (*n* = 12), and *Chlamydia pneumoniae* (*n* = 1). Percentages are expressed as detections of specified virus groups divided by all detections.

Abbreviations: Ad, adenovirus; CoV, coronavirus; Flu, influenza virus; hMPV, human metapneumovirus; PIV, parainfluenza virus; R/E, rhinovirus/enterovirus; RSV, respiratory syncytial virus.

## 4 DISCUSSION

In contrast to routine surveillance relying on MAI, ORCHARDS uses a community‐based design. Most cases of influenza do not present for medical attendance^40^ or result in hospitalization or death.^41^, ^42^ Influenza attack rates are much higher in school‐aged children than for any other demographic group,^40^ and influenza significantly contributes to school absenteeism.^43^ This is reflected by the prominence of influenza detections in ORCHARDS participants, of whom 79% reported school absenteeism. Accordingly, ORCHARDS is designed to detect and evaluate—over multiple seasons—temporal trends of influenza detection among school‐aged children who are central to community‐wide influenza transmission and who are less represented among MAI cases.

A number of studies evaluating absenteeism and influenza predate ORCHARDS^15^, ^16^, ^17^, ^18^, ^44^ but have been limited by evaluating single outbreaks. Influenza does not follow a regular pattern but rather encompasses outbreaks of variable magnitudes and temporal patterns due to differing influenza types, subtypes, and clades.^45^, ^46^ Moreover, influenza outbreaks can occur at any time over a fairly wide seasonal range,^47^ thus making assessments over several seasons necessary to evaluate for effects of timing. Finally, an observational approach allows accumulation of multiple periods of planned and unplanned (weather‐related) school breaks that may allow evaluation of school closure for outbreak response.

ORCHARDS takes advantage of a long‐standing and highly effective influenza surveillance system as a “gold standard” for daily comparability. This parallel system is based on MAI surveillance at five family medicine clinics overlapping with the study catchment area and using very similar data instruments and identical laboratory methods. The community involvement, longitudinal nature, and external comparability make ORCHARDS a unique study platform to evaluate the role of school‐aged children on influenza transmission and the utility of cause‐specific absenteeism monitoring for identifying influenza outbreaks.

## AUTHOR CONTRIBUTIONS

**Jonathan Temte:** Conceptualization; data curation; funding acquisition; investigation; methodology; supervision. **Shari Barlow:** Conceptualization; funding acquisition; methodology; project administration; resources; supervision. **Maureen Goss:** Conceptualization; data curation; investigation; methodology; project administration; validation. **Emily Temte:** Conceptualization; data curation; investigation; methodology; project administration; validation. **Cristalyne Bell:** Data curation; project administration; validation. **Cecilia He:** Data curation; project administration; validation. **Caroline Hamer:** Data curation; project administration. **Amber Schemmel:** Data curation; project administration. **Brad Maerz:** Data curation; project administration. **Lily Comp:** Data curation; project administration; validation. **Mitchell Arnold:** Data curation; project administration; validation. **Kimberly Breunig:** Data curation; project administration; validation. **Sarah Clifford:** Data curation; project administration. **Erik Reisdorf:** Conceptualization; resources. **Peter Shult:** Conceptualization; resources. **Mary Wedig:** Conceptualization; resources. **Thomas Haupt:** Conceptualization; resources. **James Conway:** Conceptualization; methodology. **Ronald Gangnon:** Formal analysis; visualization. **Ashley Fowlkes:** Conceptualization; methodology. **Amra Uzicanin:** Conceptualization; methodology.

### PEER REVIEW

The peer review history for this article is available at https://publons.com/publon/10.1111/irv.12920.

## Supporting information


**Data S1.** Supporting Information

**Data S2.** Supporting Information

## Data Availability

The datasets generated and/or analyzed during the current study are not publicly available because the study is ongoing but may be available from the corresponding author upon reasonable request.
