# Supplementary material for: The Oregon Child Absenteeism Due to Respiratory Disease Study (ORCHARDS): Rationale, objectives, and design
Source: Influenza Other Respir Viruses. 2021 Oct 8;16(2):340–50. doi: 10.1111/irv.12920 (PMC8818813; doi:10.1111/irv.12920)
Supplement: Supplementary file 2 — Data S2. Supporting Information [file IRV-16-340-s002.pdf]

ORCHARDS HOUSEHOLD STUDY FORM

School ID: ______________

Participant ID: ___________

NASAL SWAB

ID

HOUSEHOLD MEMBER NAME: ____________________

RELATIONSHIP TO STUDENT: _____________________

Age: _____ **Do you work outside the home?** Yes No **Number of bedrooms:** _____

Gender: F M **Do you attend school?** Yes No **Do you attend Daycare?** Yes No

- -

TODAY Day 0 (___/___/___)

-Did you receive an influenza vaccine this year (after August 1, 2019)? Yes No

Have you had cold or flu-like symptoms in the past 7 days? Yes No (if **No**, then you are done until next week)

If yes: How many days ago did your symptoms start? _______

How severe are/were your symptoms? Mild Moderate Severe

What symptoms have you had in the past 7 days? (circle all that have been present)

Fever	Chills	Cough	Runny Nose	Sore Throat
Tiredness	Body Aches	Headache	Poor Appetite	Nasal Congestion

Were you seen by a healthcare provider? Yes No **Where?** Usual Clinic Urgent Care ER

What diagnosis were your given? _____________________

Were you given an antibiotic or antiviral medication? Yes No _______________________

Were you sent to the hospital? Yes No

Did you miss school or Work? Yes No **If yes, how many days did you miss?** ______

- -

FOLLOW-UP Day 7 (___/___/___)

Have you had cold or flu-like symptoms in the past 7 days (since our previous visit)? Yes No

If yes: How many days ago did your symptoms start? _______

How severe are/were your symptoms? Mild Moderate Severe

What symptoms have you had in the past 7 days? (circle all that have been present)

Fever	Chills	Cough	Runny Nose	Sore Throat
Tiredness	Body Aches	Headache	Poor Appetite	Nasal Congestion

Were you seen by a healthcare provider? Yes No **Where?** Usual Clinic Urgent Care ER

What diagnosis were your given? _____________________

Were you given an antibiotic or antiviral medication? Yes No _______________________

Were you sent to the hospital? Yes No

Did you miss school or Work? Yes No **If yes, how many days did you miss?** ______
